# Recent progress in noble-metal-free electrocatalysts for alkaline oxygen evolution reaction

**DOI:** 10.3389/fchem.2022.1071274

**Published:** 2022-12-08

**Authors:** Deming Tan, Hao Xiong, Tao Zhang, Xuelin Fan, Junjie Wang, Fei Xu

**Affiliations:** ^1^ School of Mechanical Engineering, Chengdu University, Chengdu, China; ^2^ School of Materials Science and Engineering, Northwestern Polytechnical University, Xi’an, China; ^3^ Institute of Process Engineering, Chinese Academy of Sciences, Beijing, China; ^4^ Institute of Botany, Jiangsu Province and Chinese Academy of Sciences, Nanjing, China; ^5^ Jiangsu Key Laboratory for the Research and Utilization of Plant Resources, Jiangsu, China

**Keywords:** OER electrocatalyst, electrochemical water splitting, noble-metal-free electrocatalyst, water electrolysis, anion exchange membrane electrolysis

## Abstract

The practical application of splitting water to generate hydrogen is to a large extent hindered by an oxygen evolution reaction (OER) process. Electrocatalysts with low-cost, high activity, and durability are essential for the low kinetic threshold of the OER. Despite the high active performances of noble metal compound electrocatalysts like IrO_2_ and RuO_2_, they are heavily restricted by the high cost and scarcity of noble metal elements. In this context, noble-metal-free electrocatalysts have acquired increasing significance in recent years. So far, a broad spectrum of noble-metal-free electrocatalysts has been developed for improved OER performance. In this review, three types of electrolysis and some evaluation criteria are introduced, followed by recent progress in designing and synthesizing noble-metal-free alkaline OER electrocatalysts, with the classification of metal oxides/(oxy)hydroxides, carbon-based materials, and metal/carbon hybrids. Finally, perspectives are also provided on the future development of the alkaline OER on active sites and stability of electrocatalysts.

## 1 Introduction

Splitting water to generate H_2_ and O_2_, through the hydrogen evolution reaction (HER) and oxygen evolution reaction (OER), is regarded as an ideal solution to the energy shortage and climate problems. (Doyle and Lyons, 2016; Liu et al., 2018; Lyu et al., 2019) Benefitting from its high-density energy and non-polluting nature, H_2_ is usually acknowledged to be the ultimate clean energy. (Hunter et al., 2016; Zuo et al., 2019; Park et al., 2022) However, the realization of large-scale production of H_2_ is heavily dependent on the development of low-cost alternative technologies. Steam reforming of natural gas can be used for large-scale production of H_2_ (Wu et al., 2020; Ďurovič et al., 2021), but this method is unsustainable and harmful to the environment. Alternatively, the water electrolysis produces hydrogen gas in a renewable and potentially cost-effective way, which utilizes electric power to split the oxygen and hydrogen bond in a water molecule. It is an especially attractive solution for H_2_ production compared to the use of steam reforming of natural gas. (Jamesh and Harb, 2021) According to the type of ions transporting through the electrolyte and operating temperature, three main types (Plevová et al., 2021) of the water electrolysis process are distinguished: high-temperature solid oxide steam electrolysis, proton exchange membrane (PEM) electrolysis, and anion exchange membrane (AEM) electrolysis (Carmo et al., 2013; Peng et al., 2016; Reier et al., 2017; Xu et al., 2018; Todoroki and Wadayama, 2019; Li et al., 2021).

### 1.1 High-temperature solid oxide steam electrolysis

Solid oxide steam electrolysis exhibits high performance in the absence of noble-metal catalysts like platinum. Nevertheless, this is at the cost of the high energy consumption and strict material requirements, thus becoming the least-used technology among the three types mentioned in this study. (Hauch et al., 2008)

### 1.2 PEM water electrolysis

PEM electrolyzers use proton exchange membranes to separate the gas products. (Carmo et al., 2013) It is a highly flexible, intensive, and compact technology with higher current density, lower Ohmic losses, and a larger partial load range in comparison to alkaline electrolyzers. Nevertheless, the high cost of PEM and the narrow choice of available catalysts hinder its widespread application. (Eftekhari, 2017a)

### 1.3 AEM water electrolysis

**FIGURE 1:**
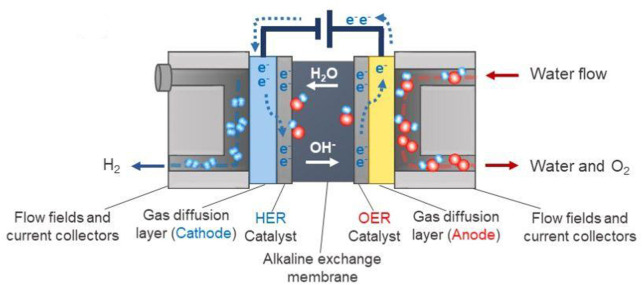
Schematic diagram of an AEM process system. (Xu et al., 2018) Copyright 2018, American Chemical Society.

In contrast to the PEM process, the AEM process performs in an alkaline environment (Figure 1), which could circumvent the limitations of the PEM system and enable the use of cheap catalysts like metal oxides. (Xu et al., 2018) Consequently, AEM is by far the most prevailing technology because of its low cost and a variety of available catalysts, making it an attractive candidate for large-scale commercial applications, e.g., in hydrogen production. (Pletcher and Li, 2011)

AEM is an attractive option because of its well-matured technology in the hydrogen economy scheme, which however AEM technology needs further improvement to become competitive with the PEM process. This goal creates some important research fields of AEM and leads to the development of increasingly more active catalysts. (Ďurovič et al., 2021) Therefore, this review specifically focuses on the catalysts for an OER in the AEM process. In the following section, fundamental electrochemistry and evaluation criteria of an OER in the AEM process will be discussed.

### 1.4 Fundamental electrochemistry of an OER in alkaline environments

Under alkaline conditions, the overall water oxidation reaction is (Liang et al., 2021)

$$4{OH}^{-}\to O_{2}\left(g\right)+2H_{2}O\left(l\right)+4e^{-},$$ (1)

where (g) refers to the gas phase and (l) the liquid phase.

The OER process in an alkaline medium is considered to proceed as the following steps (Zhou et al., 2017):

*+OH−→ OH*+e−, (2)

OH*+OH− → O*+H2Ol+e−, (3)

O*+OH−→OOH*+e−, (4)

OOH*+OH−→*+O2g+H2Ol+e−, (5)

where ^∗^ represents the catalyst active site and ^∗^OH, ^∗^O, and ^∗^OOH represent the species adsorbed on the active site.

### 1.5 Evaluation criteria of OER catalysts

It is essentially important to establish standards for evaluating the performance of OER electrocatalysts. Here, we summarized some well-established criteria including overpotential (η), onset potential, turnover frequency (TOF), Faradic efficiency, Tafel slope (b), stability, and activity. (Tahir et al., 2017; Lyu et al., 2019)

#### 1.5.1 Overpotential

The overpotential critically determines the extra energy consumption and energy conversion efficiencies. Low overpotential is a noteworthy quality of OER catalysts for reducing these parameters. (Wei and Xu, 2018)

η=b∙log⁡∣j∣+a, (6)

where

$$a=\frac{2.303∙RT}{\alpha F}\log\left(j_{o}\right),$$ (7)

$$b=\frac{-2.303∙RT}{\alpha F}.$$ (8)

Here, j represents the corresponding current density, j_o_ represents the exchange current density, and *α* represents exchange coefficients, and generally, *α* ≈ 1/2. (Naimi and Antar, 2018)

#### 1.5.2 Tafel slope

Another significant parameter is the Tafel slope (b), which can be calculated from the equation (Park et al., 2022). A high Tafel slope means a rapid increase of overpotential with the current density, indicating inferior OER kinetics of the electrocatalysts.

#### 1.5.3 Faradic efficiency

Faradic efficiency (FE) is the ratio of the amount of target gas detected in the experiment to the amount of gas calculated. (Sun et al., 2022) Faradic efficiency can be calculated as follows:

$$FE=\frac{4Fn_{O_{2}}}{It}\times 100\%,$$ (9)

where F represents Faraday’s constant, $n_{O_{2}}$ represents the amount of produced O_2_, I represents the constant current applied, and t represents the reaction time.

#### 1.5.4 Turnover frequency

The TOF for the OER is the average number of moles of O_2_ evolved per active site and time unit. (Ďurovič et al., 2021) It is also an important parameter to estimate the OER catalysts. The calculation of TOF is demonstrated as follows:

$$TOF=\frac{jS}{4nF}.$$ (10)

Here, j is the current density, S represents the electrode area, and n represents the number of active sites.

Active sites normally originate from the surface self-reconstruction during the dynamic catalysis process. (Fabbri et al., 2017) However, it is difficult to determine the precise number of active sites in practice. The evaluation of the number of active sites by measuring the total active species often leads to overestimated results as inert species are not excluded. Despite the advances in the current understanding of reaction mechanisms, the exact mechanism involved in a catalysis process is still insubstantial. A combination of various *in situ* techniques and theoretical calculations may be helpful to better understand the reaction mechanism and the true active sites.

#### 1.5.5 Electrolyte

Acidity and basicity of the electrolyte exert considerable influence on the performance of OER electrocatalysts. So far, as reported in literatures, alkaline electrolytes are the most favorable for most OER electrocatalysts, followed by neutral electrolytes, and the least favorable for acidic electrolytes. (Tahir et al., 2017; Cai et al., 2019; Zeradjanin et al., 2021; Kim et al., 2022) At present, most researchers focus on designing and synthesizing electrocatalysts which are stable in an alkaline electrolyte. Nevertheless, most of these catalysts cannot resist the high oxidative potential under acidic conditions. Therefore, it is of great importance to develop OER electrocatalysts that can perform over a wide pH range.

#### 1.5.6 Stability

It is essential to evaluate the stability of electrocatalysts toward practical applications. An electrolyte plays an important role in the stability of catalysts as most catalysts are apt to be corrupted in an acidic medium but can perform well in a basic medium. The stability of catalysts is also affected by a working electrode in most cases, and typically catalysts fabricated on the working electrode directly show higher stability. It is questionable whether the reported stability in the literature meets the requirements of real devices. The recently developed techniques which employed *ex situ* and *in situ* XRD, TEM, SEM, and XPS for monitoring the structure evolution of catalysts might be the answer to the problem.

The OER is a key step in the AEM process, but it is kinetically sluggish due to its four-electron transfer process. (Xie et al., 2022) In order to maintain the momentum of future advances of water splitting, the development of high-performance OER electrocatalysts is necessary. RuO_2_ and IrO_2_ are at present the ideal OER catalysts because of their high catalytic activity both in an acidic and alkaline environment. Nevertheless, the scarcity of Ir and Ru elements inducing the large-scale production of RuO_2_ and IrO_2_ in an OER runs into bottlenecks. (Ďurovič et al., 2021) For the last few years, the search for more abundant and lower cost alternatives to the noble-metal-based catalysts has stimulated an influx of research into this field and leads to significant advances. (Doyle and Lyons, 2016; Eftekhari, 2017b; Stelmachowski et al., 2021) For instance, some noble-metal-free-based OER electrocatalysts like transition metal-based materials (Huynh et al., 2015; Gao et al., 2021; Wang et al., 2021; Wang et al., 2022) and carbon-based materials (Gao et al., 2019) are recently reported to perform well in both acidic and alkaline conditions. The encouraging results shows that some noble-metal-free-based electrocatalysts exhibit a remarkable catalytic activity in alkaline conditions and sometimes are superior to noble-metal-based electrocatalysts (Lu et al., 2017; Lu et al., 2019). For this reason, this review aims to briefly summarize the recent advances in noble-metal-free electrocatalysts for an alkaline OER process.

## 2 Noble-metal-free electrocatalysts for the OER in an alkaline electrolyte

### 2.1 Non-precious metal oxide/(oxy)hydroxide materials

Noble-metal-free metal oxides/(oxy)hydroxides attracted great interest due to their outstanding stability and abundance (Marschall and Wang, 2014). They can be prepared and/or obtained directly from metal X-ides (X-: sulf-, nitr-, carb-, chalcogen-, etc.) during an OER process. (Ding et al., 2019; Huang et al., 2019; Zuo et al., 2019; Xie et al., 2022) In alkaline electrolytes, iron oxides/hydroxides, cobalt oxides/hydroxides, and nickel oxides/hydroxides have been studied extensively among the earth-abundant metals, which thus will be emphasized in the following.

**FIGURE 2:**
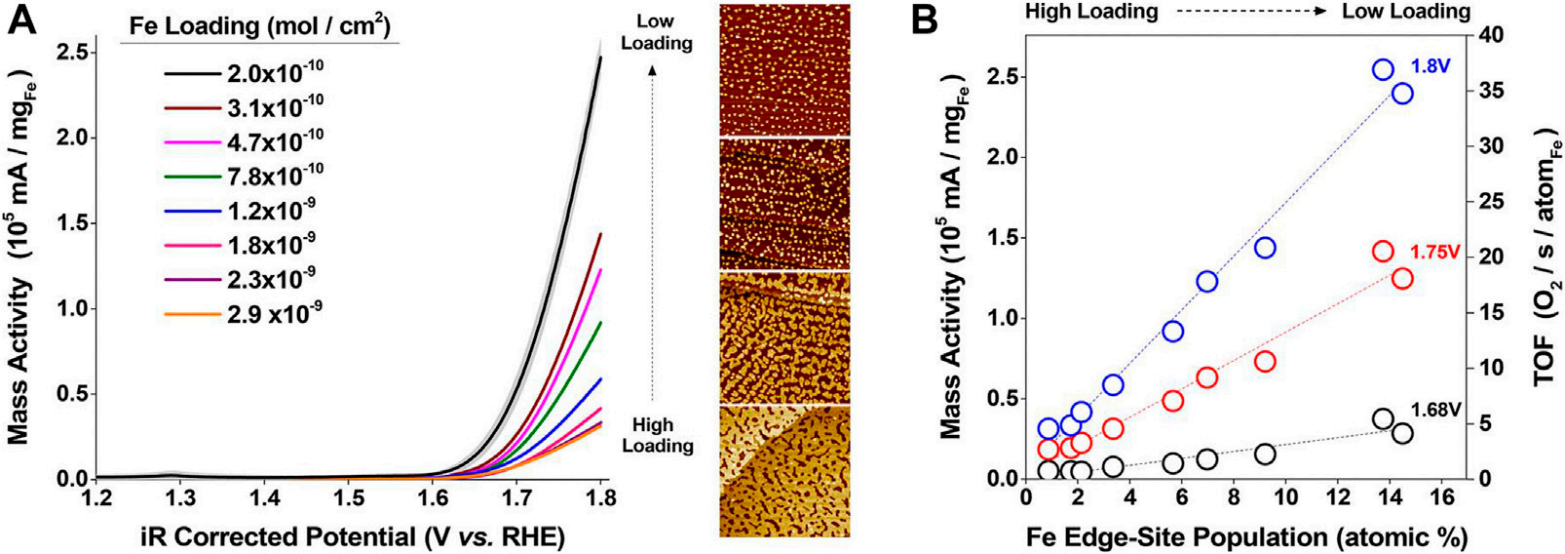
**(A)** OER voltammograms of 2L-Fe_2_O_3_/Au under different loadings in N_2_ purged 0.1 M KOH (Kauffman et al., 2019). **(B)** TOF and mass activity *versus* the relative population of edge-site located Fe atoms (Kauffman et al., 2019). Copyright 2019, American Chemical Society.

Iron oxides are promising electrocatalysts due to the low cost and abundance of iron and have been studied extensively. (Hunter et al. 2016) found that OER activity of Fe_2_O_3_ nanostructures depends on the number of Fe edge-site atoms. (Kauffman et al., 2019) Edge-site located Fe atoms were recognized as the main reaction centers, and the OER turnover frequencies produced by an edge-site located Fe is approximately 150 times greater than that of Fe atoms on the surface (Figure 2). Furthermore, by using DFT calculations, more advantageous edge site-based OERs were revealed, which have lower predicted overpotentials benefited from the modification of intermediate binding. Boettcher et al. reported a new OER catalytic model of iron (oxy) hydroxide in alkaline media. (Zou et al., 2015) The catalyst dissolution rate is low at overpotentials of about 350 mV. Mass loading influences the OER current density to a great extent, and the AuOx/Au substrate can greatly improve the catalytic activity. The choice of the substrate has little influence on the activity, and a linear relationship between the current density and loading is identified. (Zou et al., 2015) J. Xu and co-workers designed and successfully synthesized ferromagnetic Co_3_−xFe_x_O_4_ spinels using sulfurization. (Wu et al., 2021) Benefitting from limited oxyhydroxide layer and the stable configuration of Co_3_−xFe_x_O_4_/Co(Fe)O_x_H_y_, the reconstructed Co(Fe)O_x_H_y_ owns an order of magnitude higher catalytic activity than the directly synthesized Co (Fe) oxyhydroxides. (Wu et al., 2021)

**FIGURE 3:**
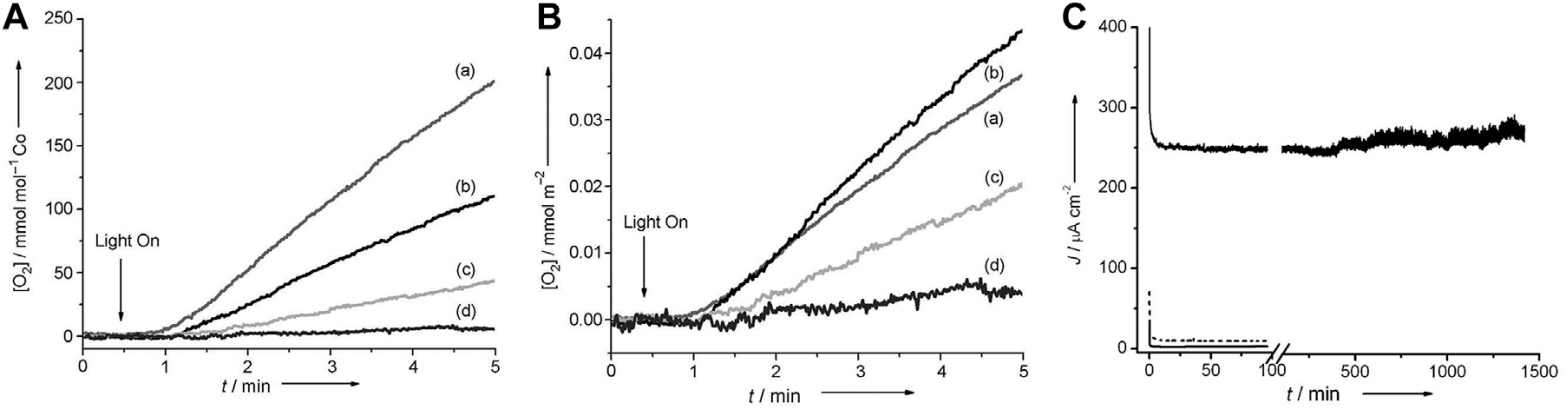
**(A)** Concentration of dissolved O_2_ measured by a Clark-type electrode at 23°C, pH 5.8 for lithium cobalt oxides synthesized at **(A)** 400°C, **(B)** 500°C, **(C)** 600°C, and **(D)** 700°C, and **(B)** the total surface area of a synthesized catalyst. **(C)** Bulk electrolysis of the Nafion-only control (solid gray line), 700-LiCoO_2_ (dashed gray line), and 400-Li_2_Co_2_O_4_ (solid black line). (Gardner et al., 2012) Copyright 2012, Wiley-VCH.

Cobalt oxides have been used as water oxidation catalysts for more than 70 years. (Hunter et al., 2016; Yu et al., 2022) Co_2_O_3_, CoO_2_, and CoO were first found feasible under anodic bias by initial electrochemical characterization in alkaline conditions. (El Wakkad and Hickling, 1950) Some newly developed structures have shown greater prospects, such as Co_3_O_4_. (Dangwal Pandey et al., 2012) Dismukes and co-workers synthesized lithium cobalt oxide having two polymorphs. The results revealed that the existence of a cubic core of Co_4_O_4_ indicates the activity of water oxidation. (Gardner et al., 2012) The synthesized pure cubic 400-Li_2_Co_2_O_4_ per bulk cobalt atom has a TOF value of 1.0 × 10^−3^ s^−1^ (Figures 3A,B). The TOF has reached 1.9 × 10^−3^ s^−1^ after normalization to the amount of cobalt that is accessible on the surface. The electrochemical characterization of membrane electrode assemblies (MEAs) reveals that the current density of the MEA containing cubic 400-Li_2_Co_2_O_4_ is 50 times higher than of the current for the MEA only with Nafion (Figure 3C). Wang et al. successfully synthesized Co_3_O_4_ nanoparticles with controllable size and found that the absence of ligands can lead to high activity. (Grzelczak et al., 2013) The tendency that the activity increases with the decrease in particle size reported in the article explicitly demonstrated that water oxidation was primarily dependent on the availability of the surface cobalt atoms. (Grzelczak et al., 2013) Müller et al. successfully synthesized composition- and size-controlled Co_3_O_4_ nanoparticles without surfactants. (Blakemore et al., 2013) The obtained cobalt oxide nanoparticles delivered the highest TOF (0.21 mol O_2_ (mol Co_surface_)^−1^ s^−1^), and the overpotential of their Co_3_O_4_ nanoparticles obtained is 314 mV at 0.5 mA cm^−2^. Meanwhile, the mass activity of 10 A m^−2^g^−1^ is achieved at 500 mV overpotential. (Blakemore et al., 2013) More recently, Yeo et al. reported an iron (III) ion-adsorbed amorphous cobalt oxide as high-efficient OER catalysts. (Gong et al., 2017) The synergetic effect of Fe^3+^ and CoO_x_ reduces the overpotential of up to 69 mV at 10 mAcm^−2^. The catalyst has a low overpotential of only 309 mV and can catalyze the OER process at 10 mAcm^−2^. The stability of the obtained catalyst was enhanced compared with that of the CoO_x_ catalyst, and the Tafel slope of the synthesized catalyst was 27.6 mVdec^−1^. (Gong et al., 2017)

**FIGURE 4:**
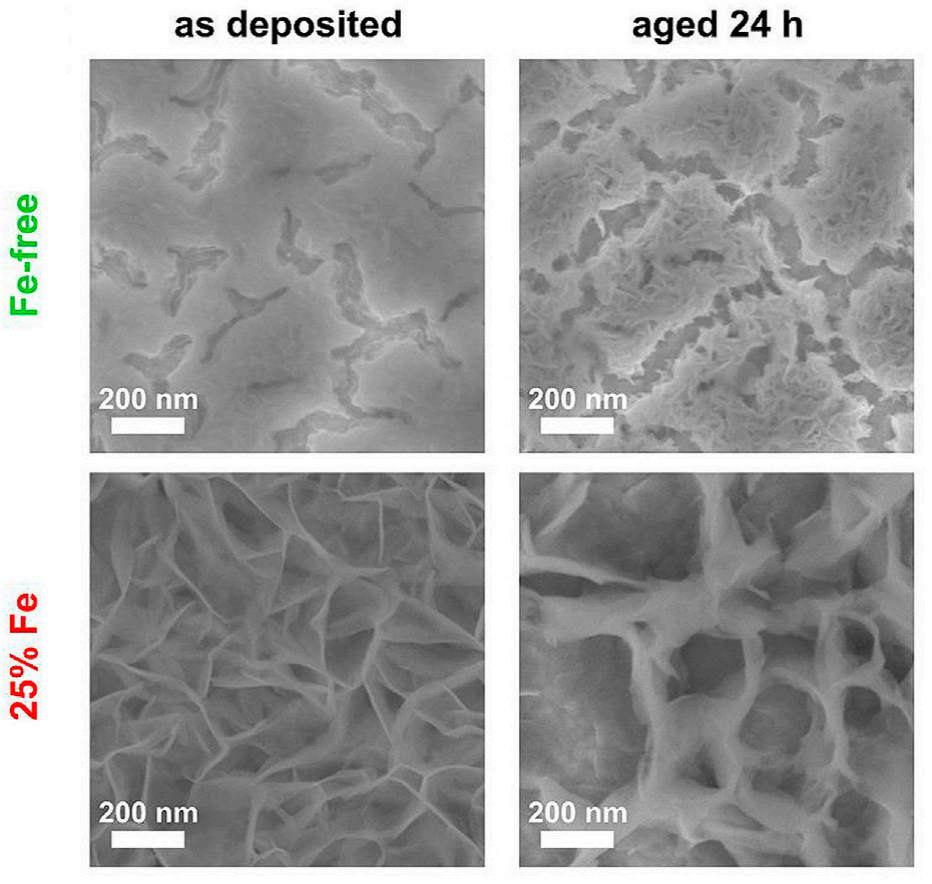
SEM images of synthesized Ni(OH)_2_ (left) and Ni_0.75_Fe_0.25_(OH)_2_ (right) catalysts as-deposited and after 24 h of aging in 40°C 1 M KOH. (Trotochaud et al., 2014) Copyright 2014, American Chemical Society.

In 1966, nickel oxides were first identified as water oxidation catalysts by Bode et al. (1966). Also, further deep studies were made in the following two decades (Oliva et al., 1982). Owing to the high OER catalytic activity in alkaline media, the application of nickel oxides in electrocatalysis has attracted great attention recently (Yu et al., 2022) (Młynarek et al., 1984). A detailed study of iron-doped nickel oxides by Boettcher et al. Trotochaud et al. (2014) underscored the essentiality of impurity influences on the performance of electrocatalysts (Figure 4). (Trotochaud et al., 2014) Lyons and co-workers investigated the mechanisms of nickel oxide electrodes and revealed the importance of Ni (III) or Ni (IV) species and proposed a more general physisorbed peroxide mechanism. (Lyons and Brandon, 2008) They used the electrode as the hole acceptor throughout instead of invoking multiple oxidation states of nickel. (Lyons and Brandon, 2008) Ma et al. reported a high catalytic active single-atom W^6+^-doped α-Ni(OH)_2_ OER electrocatalyst. (Yan et al., 2019) Overpotential values of 237 mV and 267 mV were obtained at 10 mAcm^−2^ and 80 mAcm^−2^, respectively. Also, a low Tafel slope of 33 mVdec^−1^ was obtained in a 1 M KOH electrolyte. (Yan et al., 2019)

### 2.2 Metal-free carbon-based materials

Carbon-based metal-free electrocatalysts have been considered promising OER catalysts because of their intriguing properties like nature abundance and chemical stability. (An et al., 2022) In 2009, carbon nanotubes doped with nitrogen were found to have excellent OER catalytic activity without fuel crossover and poisoning in alkaline media. (Gong et al., 2009) More recently, these new types of catalysts were discovered to be superior to metal catalysts for the OER. (Zhang et al., 2015)

**FIGURE 5:**
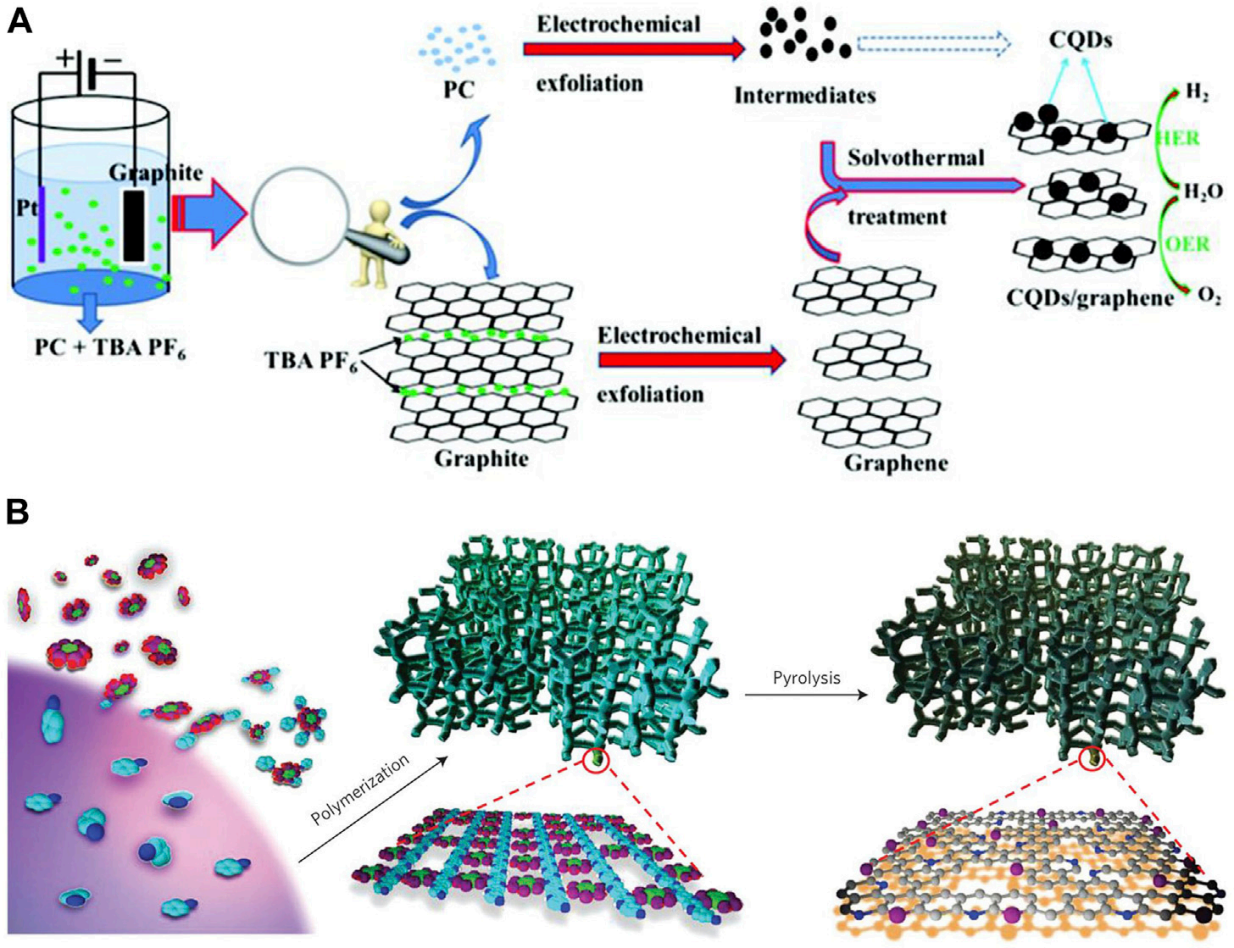
**(A)** Probable formation route of the carbon quantum dot/graphene heterostructure. (Zhao et al., 2019a) Copyright 2019, the Royal Society of Chemistry. **(B)** Preparation of porous carbon co-doped with N and P electrocatalysts. (Zhang et al., 2015) Copyright 2015, Macmillan Publishers Limited.

Dai and co-workers successfully synthesized a high-efficiency electron-acceptor by absorbing buckminsterfullerene (C_60_) onto single-walled carbon nanotubes (SWCNTs). (Gao et al., 2019) The obtained metal-free C_60_-SWCNT electrocatalysts were shown to be excellent for the OER process over a wide pH range from acidic to alkaline. (Gao et al., 2019) The current density (10 mAcm^−2^) of C_60_-SWCNT is 50, 5.3, and 1.2 times higher than that of the pure C_60_, SWCNTs and commercial RuO_2_ at 1.69 V potential, respectively. (Gao et al., 2019) Wu et al. synthesized a carbon quantum dot/graphene composite electrocatalyst with a simple novel two-step procedure (Figure 5A). (Zhao et al., 2019a) The corresponding Tafel slope of the obtained hybrid is 44 mV dec^−1^. Moreover, the stability test of obtained hybrids was carried out by CV scanning for 2000 cycles, and negligible change was observed. Such good electrocatalytic performance is attributed to various defect sites exposed by small-sized graphene flakes and carbon quantum dots, as well as the fast charge transfer rate and high active surface area. (Zhao et al., 2019a) Dai et al. reported an easy method with mass production to synthesize N and P co-doped 3D mesoporous carbon foams as bifunctional electrocatalysts for oxygen reduction and OER (Figure 5B). (Zhang et al., 2015) The obtained catalyst demonstrated lower onset potentials and higher currents than those of the Pt/C electrode. Furthermore, the material exhibits a lower onset potential than the RuO_2_ nanoparticle reference. Wang et al. successfully introduced the sp-hybridized nitrogen (sp-N) into graphdiyne, which exhibits potential for the OER. (Zhao et al., 2019b) In their research, heteroelements N and S were introduced into few-layer acetylenic groups in graphdiyne by a one-pot facile method, and the ratios of the N atoms and the S atoms were able to be tuned. (Zhao et al., 2019b) The heteroelement co-doped sample exhibits catalytic activity to those individually doped (either N or S doping) samples and most reported metal-free catalysts. (Zhao et al., 2019b) L. Xin and co-workers built a large library of single-atom catalysts by characterization and analysis of 37 monometallic elements. (Han et al., 2022) By employing an *in situ* method, they presented a unified set of principles on the state of oxidization, the number of coordinates, the length of the bond, and the coordination element of single atoms for the preparation of single-atom catalysts. They utilized the built single-atom-catalyst library to create complex multi-metallic single-atom phases with up to 12 different elements. (Han et al., 2022) The synthesized 12-metal single-atom catalyst exhibits higher current density, mass activity, and turnover frequency than those of the mixed and individual monometallic single-atom catalysts at the same potentials. (Han et al., 2022) OER catalysis of the 12-metal single-atom catalyst was performed at a constant current of 10 mAcm^−2^ for 130 h, and no appreciable potential change was observed.

### 2.3 Non-precious metal and carbon hybrids

**FIGURE 6:**
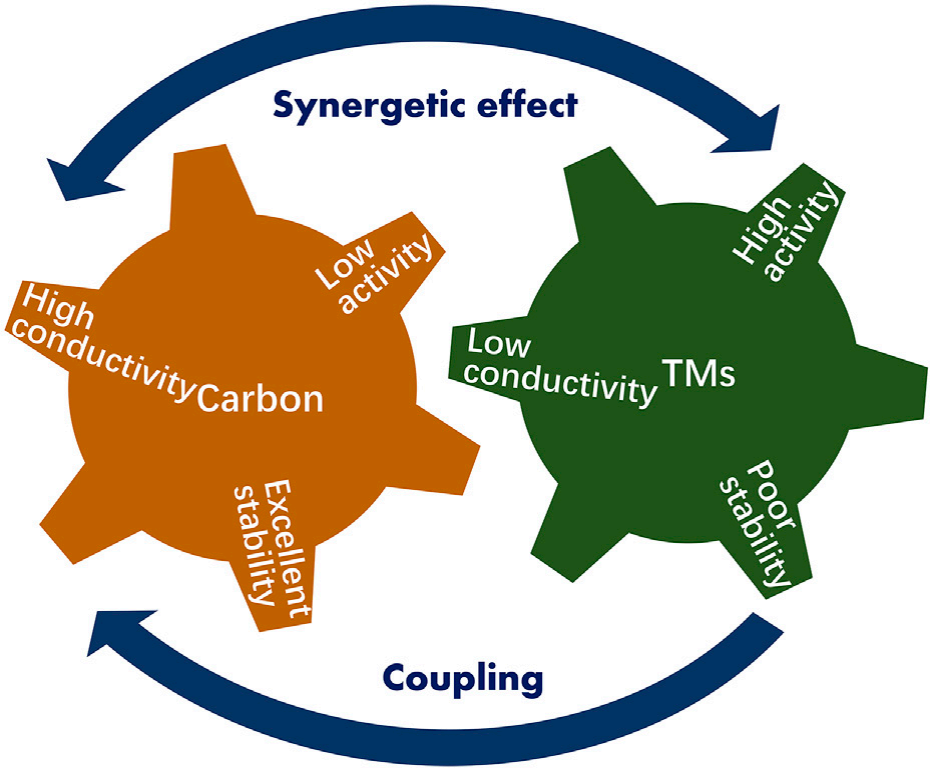
Schematic diagram of the synergistic effect of TMs and carbon nanomaterials.

The poor stability and low conductivity of transition metals (TMs) have severely impeded their use in large-scale H_2_ production. As is known, carbon nanotubes and graphites have remarkable conductivity and good chemical/thermal stability. The integration of carbon nanomaterials with TM could, therefore, circumvent the aforementioned issues on TM-based electrocatalysts (Figure 6). (Hou et al., 2015; Wang et al., 2015; Jiang et al., 2018; Hu et al., 2019) Therefore, TM/carbon hybrids are emerging as new efficient OER electrocatalysts. Usually, these composites demonstrated both the attributes of the respective components (e.g., TM or carbon) and novel promising properties achieved through the combination of carbon nanomaterials and TM, such as Ni@ nitrogen-doped graphene [Mo_3_S_13_]^2-^@ graphene. (Xu et al., 2017; Ouyang et al., 2018) The new attributes of these hybrids make them promising alternatives for the next-generation, low-cost, and efficient OER catalysts rather than a precious metal electrochemical catalyst.

Zhang and co-workers successfully achieved a mesoporous polyhedral structure of graphite carbon enclosing CoTe_2_ nanocrystals based on a single heat treatment involving tellurizing and carbonizing simultaneously. (Liu et al., 2017) The texture of the hybrid provides a simple path for the OER with increased activity and strong stability. The hybrid showed an overpotential of 300 mV at 10 mAcm^−2^. Moreover, the graphitic carbon matrix provides a high electrical conductivity and rich access to active sites besides interfacing with the confined nanocrystalline CoTe_2_ in an intensive way for the enhancement of the OER. (Liu et al., 2017) Huang and co-workers proposed a general and rational strategy for a group of monodispersed TMs embedded in graphene. (Fei et al., 2018) They have established that the resulting M@graphene (M = Fe, Co, Ni) hybrids lead to minimum distortion of the 2D graphene lattice. More importantly, the existence of the well-defined MN_4_C_4_ moieties in different holey graphene frameworks provides a desirable model system for determining and quantifying the relationship between the atomistic structure of the metal centers and its catalytic properties. Owing to the high activity of the intrinsic activity of Ni embedded in nitrogen-doped holey graphene frameworks, the density of the active sites increases as the metal loading increases. (Fei et al., 2018) Zhu et al. developed an effective bifunctional electrocatalyst based on porphyra-derived S-doped Fe-N-C. (Zhang et al., 2019) The resultant hybrid exhibits a low Tafel slope of 59 mV dec^−1^ and an overpotential of 410 mV at 10 mAcm^−2^ in a 0.1 M KOH medium. Further investigation revealed that S-doping could optimize the charge and spin distribution of the Fe–N–C hybrid, resulting in good activities in the OER, which mainly attributes to the Fe-Nx and Fe-N_3_|S sites individually. (Zhang et al., 2019) G. Gomes and co-workers designed an easy one-step sustainable protocol to synthesize nickel/nickel oxide@carbon bifunctional electrocatalysts from the wastes of cauliflower leaves. The electrocatalytic performance of the resultant hybrid was found dependent on the pyrolysis temperature. (Hoang et al., 2020) At 10 mA cm^−2^, the obtained hybrids require overpotentials of 346 mV and moderate Tafel slopes of 70 mV dec^−1^ for an OER in a 0.1 M KOH electrolyte. The synthesized electrocatalyst needs only 1.688 V to reach a current density of 10 mA cm^−2^ when incorporated into a two-electrode water electrolyzer. Medeiros et al. reported on the synthesis, structural, morphological, and electrochemical characterization of Ni-NiO/carbon nanofibers for an OER catalytic process by solution blow spinning. (Silva et al., 2021) The XPS analysis demonstrated that a small fraction of the species of nickel present in the fiber surface is sufficient to promote a good performance in the OER process. The performance of the Ni-NiO/C_An electrode was not affected although the size of the nanoparticles slightly exceeds that of the electrode. (Silva et al., 2021) Also, the active species of Ni^3+^ were found to be the main factor affecting the catalysis performance.

## 3 Summary and perspectives

It is essential to identify the active sites and their coordination structure for OER catalysts as those attributes are closely associated with the catalyst activity and stability. Several major types of active sites are classified: defects, electron deficient site, metal center, etc. The debate over the active site of electrocatalysts underlines the complication of recognizing the real active sites despite various experimental techniques and computational methods being employed. One possibility is to use an *in situ* technique to monitor catalyst evolution during the OER to provide significant insight into active sites.

Material instability and operational instability are considered the two main types of catalyst instability that occurred in the catalytic system and have great influences on the stable catalytic action. Material instability is usually caused by material corrosion regardless of the test conditions. Future OER catalyst applications require the development of materials that can be operated under severe working conditions. For metal oxides, material stability can be improved through structure design and composition adjustment. For carbon-based catalysts, material stability can possibly be improved by increasing the degree of graphitization. Operational instability arises during electrochemical tests and is usually caused by potential-induced element dissolution and/or structure damage. One possible way to increase the operational instability is to use lower operation potential. However, low operation potential needs substantial improvements in the catalyst activity; hence, the design of a high active OER is highly desirable for the future commercial development of water electrolysis.
